# Relational perceptions in high school physical education: teacher- and peer-related predictors of female students’ motivation, behavioral engagement, and social anxiety

**DOI:** 10.3389/fpsyg.2015.00850

**Published:** 2015-06-22

**Authors:** Felicity Gairns, Peter R. Whipp, Ben Jackson

**Affiliations:** School of Sport Science, Exercise and Health, The University of Western Australia, PerthWA, Australia

**Keywords:** need satisfaction, need support, relational efficacy, RISE, SDT

## Abstract

Although researchers have demonstrated the importance of interpersonal processes in school-based physical education (PE), there have been calls for further studies that account for multiple relational perspectives and provide a more holistic understanding of students’ relational perceptions. Guided by principles outlined within self-determination theory and the tripartite efficacy model, our aim was to explore the ways in which students’ perceptions about their teacher and classmates directly and/or indirectly predicted motivation, anxiety, and engagement in PE. A total of 374 female high-school students reported the extent to which their teachers and classmates independently (a) engaged in relatedness-supportive behaviors, (b) satisfied their need for relatedness, and (c) were confident in their ability in PE (i.e., relation-inferred self-efficacy). Students also rated their motivation and anxiety regarding PE, and teachers provided ratings of in-class behavioral engagement for each student. Analyses demonstrated support for the predictive properties of both teacher- and peer-focused perceptions. Students largely reported more positive motivational orientations when they held favorable perceptions regarding their teacher and peers, and autonomous motivation was in turn positively related to behavioral engagement ratings. These findings offer novel insight into the network of interpersonal appraisals that directly and indirectly underpins important in-class outcomes in PE.

## Introduction

Despite the health-enhancing effects of regular physical activity, adolescent participation rates typically fall below recommended guidelines (World Health Organization, 2013). In order to identify theory-driven strategies that promote youth physical activity participation, one sustained area of research has targeted youngsters’ experiences in school-based physical education (PE; e.g., Stratton et al., 2008). The emphasis on PE has developed, in part, because almost all youth access formalized PE at school, and therefore, PE acts as a vehicle through which many children and adolescents first engage with a range of sport/exercise activities. In addition, there are a number of acute benefits that may be derived through physical activity involvement (e.g., through PE) during childhood and adolescence, including elevated academic performance (e.g., Ardoy et al., 2014), enhanced physical fitness (e.g., Sallis et al., 1997), and improved self-esteem (e.g., Tremblay et al., 2000). For these reasons, researchers have devoted continued attention toward identifying the factors that contribute to, and are influenced by, individuals’ motivation and engagement in school-based PE. Students’ relations with, and perceptions about, their teachers (e.g., Bourne et al., 2015) and classmates (e.g., Cox et al., 2009; Cox and Ullrich-French, 2010) represent one group of antecedents that are important in shaping individuals’ PE experiences (e.g., engagement, motivation; see Ntoumanis, 2012), and it is this relational perspective upon which we focus our attention in this investigation.

### Conceptual Overview

A number of investigators have explored the role of social agents in PE through the lens of *self-determination theory* (SDT; for an overview, see Ryan and Deci, 2008). It is proposed within SDT that individuals may be motivated to pursue an activity due to substantively different reasons (or motives). According to SDT, individuals may participate in an activity due to relatively self-determined, or autonomous, motives (e.g., fun, interest, value), and/or due to relatively more controlled motives (e.g., coercion, reward, internal or external pressure). Intrinsic motivation represents the most self-determined form of motivation, and refers to pursuing an activity solely due to the inherent interest and enjoyment that it provides. Aside from intrinsic motivation, individuals may endorse a number of different forms of extrinsic motivation. From most to least autonomous, these dimensions are termed integrated regulation (e.g., when an activity is consistent with one’s identity), identified regulation (e.g., when an activity provides personally important outcomes), introjected regulation (e.g., when internal pressures such as guilt and shame accompany non-participation), and external regulation (e.g., participating due to external reward or coercion). Finally, individuals may also experience amotivation, which refers to an absence of motivation.

According to theory and research, autonomous motives (i.e., intrinsic motivation, integrated regulation, identified regulation) tend to support adaptive outcomes including effort, persistence, and well-being (see Ntoumanis, 2012; Standage and Ryan, 2012), and are therefore viewed as being more desirable than controlled forms of regulation (i.e., introjected regulation, external regulation). SDT also posits that in order to encourage autonomous motivation, it is important that three basic psychological needs are satisfied (Ryan and Deci, 2008). The need for autonomy represents one’s desire for choice and a sense of agency or volition regarding one’s pursuits. The need for competence reflects one’s desire to feel capable with respect to one’s actions and environment, and the need for relatedness refers to the desire to feel connected to, and understood by, significant others. There is compelling empirical evidence that when individuals feel that their needs are satisfied, they display greater self-determined (relative to controlled) motivation, which in turn promotes desirable achievement behavior (see Standage and Ryan, 2012). SDT-based research has also provided extensive insight into the instructional styles that provide support for the fulfillment of students’ needs in PE (see Cheon et al., 2012; Ntoumanis, 2012). Although the majority of this work has explored autonomy-supportive practices among teachers (for support, see Barkoukis et al., 2010; Standage and Emm, 2014), we focused our attention in this investigation on the lesser-studied implications associated with interpersonally involving, or relatedness-supportive environments (i.e., inclusive, supportive social interactions) in PE.

Within PE, there are two distinct ‘social agents’ through which individuals may derive relatedness support; that is, relatedness-supportive behaviors may be provided by one’s teacher and/or one’s peers/classmates. Teacher and peer relations have often been studied in isolation with SDT-based work (i.e., only one focal agent has been examined within a given investigation); collectively, though, the literature in this area demonstrates that perceptions of supportive behaviors (e.g., caring, showing interest) from both of these sources may contribute to students’ relatedness need satisfaction and/or motivation. With respect to teachers, for example, favorable perceptions of support have been shown to align directly or indirectly with adaptive motivational outcomes (e.g., Cox and Williams, 2008; Jackson et al., 2013). Similarly, although little attention has been directed specifically toward peer-derived relatedness support in PE, investigations focusing broadly on peer relations have highlighted that general support from one’s classmates aligns with greater autonomous motivation and enjoyment, and reduced anxiety within PE (e.g., Cox et al., 2009, 2011).

Aside from SDT-based work, a limited number of studies have also explored interpersonal influences in PE from the perspective of Lent and Lopez’s (2002) *tripartite efficacy model*. Drawing from the self-efficacy (Bandura, 1997) and interpersonal perception (e.g., Kenny and DePaulo, 1993) literatures, Lent and Lopez (2002) articulated that within interactive and instructional scenarios, individuals develop ‘relational efficacy’ beliefs that exist alongside their confidence in their own ability (i.e., their self-efficacy). The importance of students’ self-efficacy in PE is well-established (e.g., Gao et al., 2009); however, less is known about the relational efficacy appraisals that students hold in their PE classes. With particular relevance for this investigation, Lent and Lopez (2002) described that when interacting alongside/under others, individuals develop estimations regarding the confidence that those other people have in their ability. This construct, termed *relation-inferred self-efficacy* (RISE), represents a metaperception pertaining to individuals’ appraisals of another’s (or others’) confidence in their ability. Accordingly, PE students might, for example, make appraisals regarding the extent to which their teacher (e.g., “I think my teacher really believes in me”) and/or classmates (e.g., “my classmates think I’m good at PE”) are confident in their ability.

Lent and Lopez (2002) contended that individuals derive a sense of reinforcement by believing that others are confident in their ability (i.e., favorable RISE appraisals), which may account for a range of desirable outcomes, including enhanced motivation, positive affective responses, closer relational alliances, improved coping resources, and increased perceptions of support. Consistent with the tenets of the framework, preliminary evidence from research conducted within PE indicates that favorable RISE beliefs regarding one’s teacher align positively with in-class motivation (e.g., Jackson et al., 2012). In addition, it has been shown that students believe that their teacher is highly confident in their ability when they report that their teacher makes use of transformational teaching practices (Bourne et al., 2015), and utilizes relatedness-supportive teaching methods (Jackson et al., 2013). Jackson et al. (2013) for example, demonstrated that students felt that their teacher was confident in their (i.e., the student’s) ability when they believed that their teacher employed supportive, encouraging, and friendly behaviors. To date, work within PE regarding RISE has focused primarily on students’ estimations of their teacher’s confidence in their ability (Jackson et al., 2012, 2013; Bourne et al., 2015); however, recent work within undergraduate physical activity classes indicates that students may also experience positive affect and favorable perceptions of competence when they believe that their classmates, as a whole, are confident in their ability (Jackson et al., 2014).

### The Present Study

Studies couched in SDT and the tripartite efficacy framework have underscored the importance of relatedness support and RISE inferences within PE. However, the majority of SDT-based and tripartite efficacy work in PE has focused exclusively on students’ relations with and perceptions about, their teachers (e.g., teacher-provided relatedness support, teacher-focused RISE). That being the case, there are only a limited number of studies that adopt a more holistic approach and account for students’ perceptions about their peers/classmates *alongside* their teachers (for examples, see Cox et al., 2009, 2011; Hagger et al., 2009; Cox and Ullrich-French, 2010; Jackson et al., 2014). With respect to SDT, the focus on teacher-related inferences has resulted in relatively little being known about students’ perceptions of relatedness support specifically regarding their peers/classmates. Similarly, although the emphasis on teacher-related RISE perceptions is understandable given the teacher’s position of authority within the classroom, little is known about the way in which students appraise how confident their classmates (as a whole) are in their ability in PE (e.g., “my classmates don’t seem to think I’m very good at PE”). From an ecological perspective, simultaneously accounting for teacher- and peer-related perceptions may provide a more faithful representation of the social environment that exists within PE, by acknowledging the different interaction patterns that occur in this context (see Standage and Emm, 2014).

**FIGURE 1:**
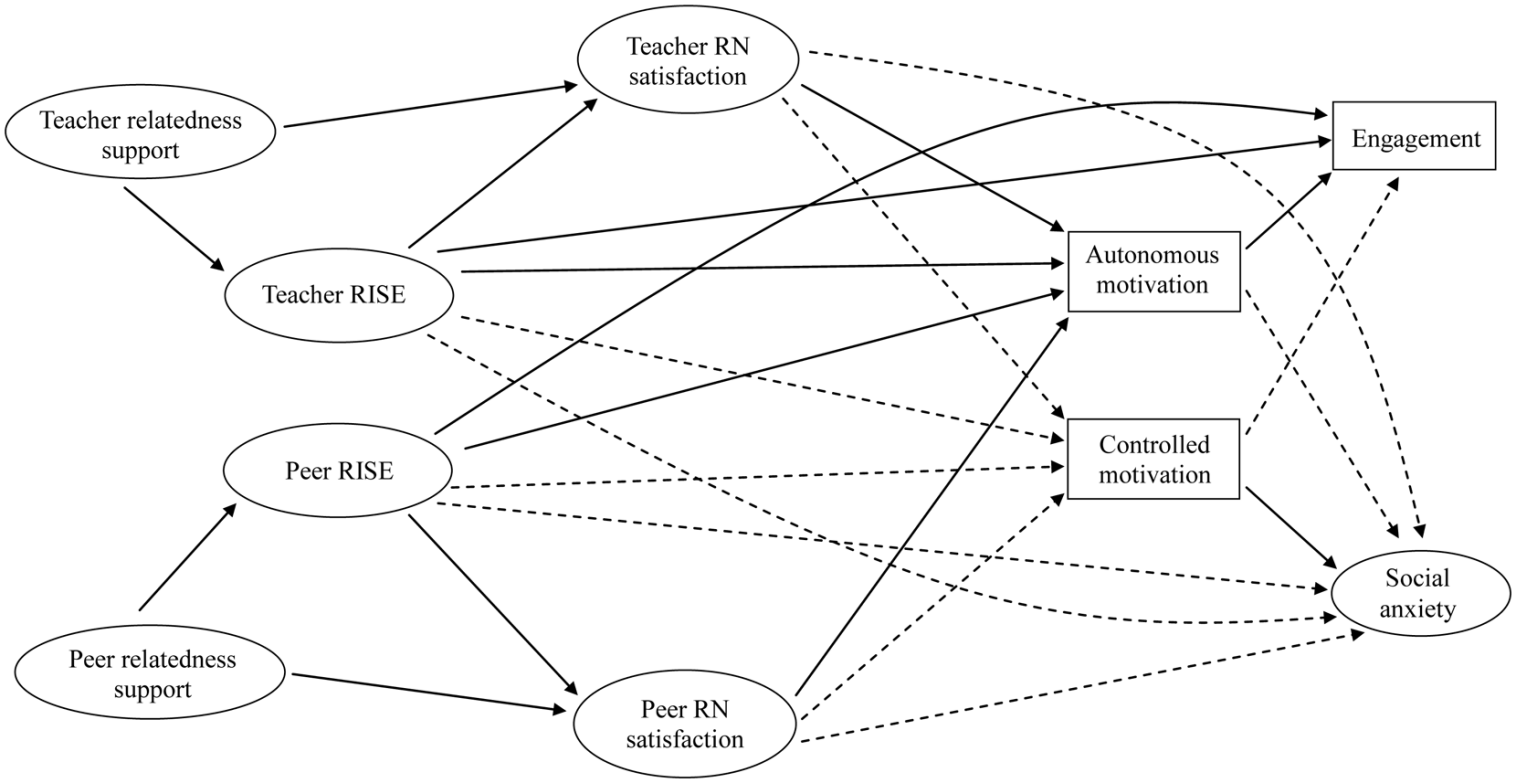
**Illustration of proposed structural model.** RN satisfaction = relatedness need satisfaction. Solid line indicates pathway was hypothesized to be positive in nature. Dashed line indicates that the relationship was hypothesized to be negative in nature. As is described in the text, in some instances (i.e., RISE → controlled motivation) there was insufficient empirical support for a firm directional hypothesies, and so our a priori hypotheses was that these variables may be either unrelated *or* related in a specific direction. For ease of interpretation, these relationships are simply indicated in terms of their directional component in this figure.

Guided by SDT and the tripartite efficacy model, our overarching aim was to examine whether PE students’ teacher- and peer-related perceptions were independently predictive of important in-class outcomes. From an SDT perspective, we sought to separate students’ impressions of teacher- and peer-derived relatedness support, as well as measuring relatedness need satisfaction pertaining to each of these agents. In terms of RISE, alongside students’ estimations regarding their teacher’s confidence in their ability (i.e., teacher-focused RISE), we also accounted for students’ estimations regarding the extent to which their classmates believed in their ability (i.e., peer-focused RISE). Accordingly, we aimed to extend the literature on relational processes in PE by (a) examining a broader network of theory-driven interpersonal appraisals, (b) exploring the extent to which respective teacher- and peer-focused perceptions were empirically distinguishable from one another, and (c) investigating how teacher- and peer-focused perceptions might directly and indirectly predict motivational, affective, and behavioral outcomes. In the following section, we provide theoretical and empirical support for the predictive pathways that we specified within our model. For the purpose of illustration, all pathways that are described in the remainder of the introduction are displayed in **Figure 1**.

In line with extant teacher-based tripartite efficacy research (Jackson et al., 2013), and Lent and Lopez’s (2002) original proposals, we hypothesized that when students perceived that their teacher and peers created a highly relatedness-supportive environment, this would predict higher levels of teacher- and peer-focused RISE, respectively. That is, when teachers (peers) were perceived to engage in behaviors that were deemed as attentive, supportive, and trusting (i.e. interpersonally involving), students would use these cues to infer that their teacher was (peers were) highly confident in their ability. We also anticipated that favorable teacher-/peer-based relatedness support perceptions would positively predict greater teacher- and peer-based relatedness need satisfaction, respectively (cf. Ryan and Deci, 2008). Aside from being predicted by relatedness support perceptions, we hypothesized that individuals would also report greater teacher- and peer-based relatedness need satisfaction when they believed that their teacher and peers (respectively) were highly confident in their ability. Although this relationship has not been empirically verified previously, we specified these pathways on the basis of Lent and Lopez’s (2002) proposal that strong RISE beliefs should promote more inclusionary and cohesive relational perceptions (i.e., feelings of closeness, support, trust).

In turn, we specified a range of predictive pathways between interpersonal (i.e., teacher-/peer-related) perceptions and students’ motivation for PE. In order to obtain a global appraisal of the quality of an individual’s motivation in a given context (e.g., PE), researchers have often previously relied on computing a single index (termed the relative autonomy index), whereby the strength of one’s autonomous motivation is weighted against the strength of one’s controlled motivation. Recently though, criticisms underlying the computation of this index have resulted in calls for alternative approaches (see Chemolli and Gagné, 2014), such as the calculation of separate indexes of autonomous and controlled motivation. With that in mind, we estimated autonomous and controlled motivation variables separately in our model (see Measures for computational information), and sought to explore relations between students’ interpersonal perceptions and both of these motivation indices. Lent and Lopez (2002) contended that favorable RISE beliefs should act as an energizing force, and should also foster enjoyment and interest in one’s pursuits. Accordingly, we anticipated that teacher- and peer-focused RISE perceptions would be directly and positively related to autonomous motivation, and would either be unrelated or negatively related to controlled motivation (cf. Lent and Lopez, 2002). Consistent with SDT tenets emphasizing the internalization function of relatedness need satisfaction (Ryan and Deci, 2000, 2008), we also hypothesized that heightened teacher- and peer-based relatedness need satisfaction would predict greater autonomous motivation toward PE, and would be negatively related to controlled motivation (cf. Standage et al., 2005).

We were subsequently guided by a range of conceptual, empirical, and practical considerations when selecting two in-class outcomes, neither of which had been previously examined in relation to this range of predictors. First, in light of the interpersonal nature of our predictor variables, we examined students’ anxiety regarding the way in which their classmates and teacher viewed/evaluated them within PE (i.e., their social anxiety). Adolescence is a developmental period during which time evaluative concerns are particularly heightened (see, for example, La Greca and Harrison, 2005), and given the public nature of performance within PE, anxiety regarding one’s appearance and competence may be highly salient. For females in particular, changes that accompany puberty have been shown to give rise to body dissatisfaction (Levine and Smolack, 2002) and social physique anxiety (SPA; Hart et al., 1989), and so we focused our attention specifically toward PE-based social anxiety perceptions among females. Guided by previous research that has demonstrated the adaptive affective processes that accompany autonomous motivation (e.g., Ntoumanis, 2005; Cox et al., 2009), we anticipated that greater autonomous motivation for PE would align with lower levels of social anxiety. In terms of controlled motivation, researchers conducting SDT-based work within PE have previously described the potential for negative affective properties associated with external regulation (e.g., Standage et al., 2005), and this construct was the primary contributor to our controlled motivation index. Moreover, students who strongly endorse external or introjected motives are driven by concerns relating to guilt, shame, and external pressures, and so intuitively it might be expected that these individuals may be more prone to apprehension regarding their participation in PE. That being the case, we hypothesized that scores on the controlled motivation variable would be positively related to students’ social anxiety. Aside from motivational variables, previous work has also demonstrated the desirable affective properties associated with students’ relatedness need satisfaction (e.g., Cox et al., 2009) and RISE beliefs (e.g., Jackson et al., 2012, 2014), and so we hypothesized that favorable perceptions on both types of relatedness need satisfaction and RISE perceptions would directly predict lower evaluative concerns.

Alongside students’ affective responses, we also sought to obtain a measure of behavioral engagement in order to identify direct and/or indirect relations between relational/motivational processes and the intensity of students’ effort in PE. Student engagement is theorized to underpin achievement and protect against drop out, and is multifaceted in nature, comprising dimensions relating to behavioral (i.e., conduct, involvement, effort), emotional (i.e., affective processes), and cognitive (i.e., comprehension, self-regulation) factors (for a review, see Fredricks et al., 2004). Within PE, it is acknowledged that engagement levels among females are often lower than among their male counterparts (e.g., McKenzie et al., 2000), and this consideration supported our focus on the way in which female students’ interpersonal perceptions may be important in predicting their engagement. In doing so, we aimed to avoid relying solely on self-report data by obtaining external (i.e., teacher) ratings of student engagement (cf. Ntoumanis, 2005), and as a result, we restricted our assessment of engagement solely to the behavioral dimension. In comparison to emotional and cognitive dimensions, behavioral engagement – in light of being overtly observable – has been shown to be particularly suited to being assessed through teacher reports (see Fredricks et al., 2004). We hypothesized that students who strongly endorsed autonomous motives for participation in PE would be rated as displaying high levels of engagement (Ntoumanis, 2005; Taylor et al., 2010). Ntoumanis (2001) also demonstrated that controlled motives predicted greater boredom (i.e., a lack of engagement), and so we also anticipated that higher scores for controlled motivation would align with lower behavioral engagement ratings. Finally, we drew from Lent and Lopez’s (2002) proposals regarding the energizing properties associated with positive RISE appraisals, and specified predictive pathways between RISE beliefs and engagement (i.e., insofar as students would display enhanced engagement when they believed that their teacher and classmates were highly confident in their ability).

## Materials and Methods

### Participants

The sample consisted of 374 female students (*Mage* = 13.36, SD = 1.19, range = 12–16) recruited from 19 separate classes within one Western Australian independent all girls’ school. Participants were drawn from grade 7 (six classes; *n* = 135), 8 (four classes; *n* = 85), 9 (four classes; *n* = 78), and 10 (five classes; *n* = 76). On average, students in grades 7 and 8 received 1.73 h of in-school PE per week, while those in grades 9 and 10 participated in 1.15 h of in-school PE per week.

### Procedure

Having received ethical approval, information sheets were provided to the principal of an all-girls independent school. Upon receiving consent from the principal, information sheets were also sent to prospective teachers, students, and parents/guardians, in which the purpose, design, and procedure of the study was described. All PE teachers of grade 7–10 students were invited to participate, and classes taught by these teachers were subsequently selected at random. Suitable times were then arranged to visit the school, and at the beginning of data collection, students were informed verbally and in writing that they could refuse to answer any questions, had the right to withdraw at any time, that all information would remain confidential, and that their peers/teachers had no influence whatsoever on their decision to participate. Informed consent was also sought from all students and teachers at the beginning of all data collection sessions. In order to avoid over-burdening participants, and to enable us to control for baseline physical activity levels, data were collected at two different occasions, with an intervening period of ∼1 month between collections. At time one, participants provided demographic data and reported their leisure-time physical activity (LTPA) over the previous week. Having completed the questionnaire, students were provided with a parent information letter and stamped addressed envelope to take home, which instructed parents to return the letter should they wish to withdraw their daughter from the study. At time two, participants completed measures of all primary variables. Given the number of measures included within the time two assessment, measurement of primary variables was split across three different time points. At the beginning of students’ first PE lesson in a specific week, measures of teacher- and peer-focused relatedness support, along with teacher- and peer-focused RISE, were completed. Another battery of measures comprising teacher- and peer-focused relatedness need satisfaction, motivation, and social anxiety was administered at the end of the second (and final) PE lesson in the same week. At the end of the second lesson, teachers also completed ratings of student behavioral engagement, referring to students’ participation in PE during that week (i.e., the period during which the student assessments were made).

### Measures

#### Perceived Relatedness Support

Students’ perceptions of teacher- and peer-focused relatedness support (i.e., the degree to which students perceived their teacher/classmates displayed interpersonally involving behaviors) were each measured with a five-item instrument (Standage et al., 2005). Using the stem, ‘At the moment, in my PE class…,’ students were asked to respond to five statements about their teacher (e.g., “my PE teacher supports me”), using a response scale anchored at 1 (*strongly disagree*) and 7 (*strongly agree*). In order to measure students’ perceptions of peer-based relatedness support, modifications were made to these five items (e.g., “my classmates have respect for me”). Previous work with similar-aged students has demonstrated support for the factorial and predictive validity of measures derived from this instrument (e.g., Standage et al., 2005). The teacher- (ρ = 0.94) and peer-focused (ρ = 0.93) measures derived from this instrument displayed an acceptable composite reliability estimate (Raykov, 1997) in this investigation.

#### Relation-Inferred Self-Efficacy

Teacher- and peer-focused RISE appraisals were each assessed using a nine-item instrument designed for use among high-school students (Jackson et al., 2012). In order to measure teacher-focused RISE, respondents were instructed to think about their PE class and estimate “right at this moment in time, how confident do you think your PE teacher is in your ability to…,” followed by a series of statements including, “try your hardest in every PE class,” and “perform all the skills you are taught in PE.” To ensure understanding, a further statement was included, “we’re not focusing on how confident you are; we’re focusing on whether you think your PE teacher is confident in you or not.” In order to measure peer-focused RISE, modifications were made to instructions, including “right at this moment in time, how confident do you think your peers are in your ability to…,” and “we’re not focusing on how confident you are; we’re focusing on whether you think your classmates, as a whole, are confident in you or not.” Responses were made on a 5-point scale anchored at 1 (*no confidence at all*) and 5 (*complete confidence*). The internal consistency and validity of measures derived from this instrument have previously been demonstrated with similar-aged students (Jackson et al., 2012), and an acceptable level of internal consistency was observed for the teacher- (ρ = 0.89) and peer-focused (ρ = 0.91) measures derived from this instrument.

#### Relatedness Need Satisfaction

Student perceptions of teacher- and peer-focused relatedness need satisfaction were each assessed using Richer and Vallerand’s (1998) five-item instrument. For teacher-related perceptions, the generic ‘PE-focused’ stem was modified to read, “With my teacher in this PE class I feel…,” and five items (e.g., “supported,” “listened to”) were rated on a 7-point scale ranging from 1 (*strongly disagree*) to 7 (*strongly agree*). For peer-based perceptions, instructions were modified to, “With my peers in this PE class I feel…” Previous PE investigations have demonstrated support for the psychometric properties of measures derived from this instrument (e.g., Standage et al., 2003); we observed acceptable internal consistency for measures derived from teacher- (ρ = 0.94) and peer-based (ρ = 0.94) instruments.

#### Motivation

Students’ motivation for PE was measured using the Perceived Locus of Causality (PLOC) scale (Goudas et al., 1994). Following the stem, “at the moment, I take part in PE classes…,” students responded to statements that assessed intrinsic motivation (four items; e.g., “because I enjoy learning new skills”), identified regulation (four items; e.g., “because I want to learn sport skills”), introjected regulation (four items; e.g., “because I want the teacher to think I’m a good student”), external motivation (four items; e.g., “because that’s what I’m supposed to do”), and amotivation (four items; e.g., “but I don’t really know why”). Students responded to each item on a 7-point scale ranging from 1 (*strongly disagree*) to 7 (*strongly agree*). Considerable research in PE has demonstrated support for the psychometric properties for measures derived from the PLOC scale (e.g., Lonsdale et al., 2011). For analysis purposes, we created two observed variables; one that reflected students’ controlled motivation (i.e., using a weighting formula; 2 × external regulation; 1 × introjected regulation), and one that reflected their autonomous motivation (i.e., using a comparable weighting formula; 2 × intrinsic motivation; 1 × identified regulation).

#### Social Anxiety

In line with previous research (Martin and Fox, 2001), participants’ concerns regarding their teacher’s and classmates’ impressions of them during their PE lessons were each measured using four items. Minor revisions were made to Martin and Fox’s (2001) instrument (i.e., the term ‘instructor’ was changed to ‘teacher,’ and ‘participants’ was changed to ‘classmates’); students responded to four items about their teacher, before completing the same four items with respect to their classmates (e.g., “I am concerned about looking uncoordinated in front of my teacher/classmates,” “I worry about embarrassing myself in front of my teacher/classmates”) using the stem, “thinking about how I feel in my current PE lessons…” Responses were made on a 5-point scale ranging from 1 (*not at all concerned*) to 5 (*extreme concern*). Martin and Fox presented evidence to support the internal consistency of measures derived from this instrument, and we observed acceptable internal consistency for a combined teacher-and-peer measure derived from this instrument (ρ = 0.93^1^).

#### Behavioral Engagement

Teachers responded to a single item regarding students’ in-class behavioral engagement (i.e., “over this week, what level of engagement has this student shown in your PE class?”). Teachers rated each student on a 7-point scale, anchored at 1 (*no engagement*), 4 (*average engagement*), and 7 (*very high level of engagement*) based on the intensity of their participation (relative to their classmates) within the last week. A single item was used at the request of teachers in order to minimize response burden; similar approaches have been implemented previously for the measurement of behavioral engagement, and have been shown to display evidence of criterion validity (e.g., Ntoumanis, 2005).

#### Leisure-Time Physical Activity

Students’ LTPA levels were measured (for use as a covariate in our main analyses) using the Leisure-Time Exercise Questionnaire (LTEQ; Godin and Shephard, 1985). Definitions and examples for mild, moderate, and vigorous activity categories were provided, and all students reported bouts of mild, moderate, and vigorous physical activity (>20 min) that they had completed over the previous week. Students were asked to exclude any curriculum-based (e.g., PE) activity, as well as any other compulsory school-based physical activity. Godin and Shephard’s (1985) formula (i.e., 9 × number of vigorous bouts + 5 × number of moderate bouts + 3 × number of mild bouts) was used to calculate an LTPA score. Support has been demonstrated for the psychometric properties of the LTEQ with samples similar to those within the present investigation (e.g., Hagger et al., 2005).

#### Data Analysis

First, we examined item-level descriptive statistics in order to determine distributional properties and to screen for outliers. We then estimated a structural equation model incorporating all measurement parameters and structural pathways in Mplus version 7.11 (Muthén and Muthén, 1998–2013). Given that students were nested within classes, we implemented a correction for non-independence of observations based on student clustering (Asparouhov and Muthén, 2006). Missing data were treated using a full information maximum likelihood method, and we used a robust maximum likelihood estimator (MLR), which creates SE that are robust to any deviation from normality, and to the use of categorical indicators that comprise five or more response categories (e.g., Rhemtulla et al., 2012). We specified a single model that included all direct and indirect pathways between latent and observed variables. Each latent variable was specified using all the items/indicators for that variable (see Measures for number of items per latent variable). We also modeled grade level and LTPA as single-item covariates in order to control for their potential effects on all endogenous variables.

Given the lack of consensus regarding the suitability of different fit indices in making firm conclusions regarding model fit (e.g., Marsh, 2007), we implemented a multi-faceted approach when optimizing and judging model fit. In particular, as well as generating models that were consistent with theory, we utilized modification indices to address potential misfit in our initial specification, and followed recommendations (Hu and Bentler, 1999; Byrne, 2012) by considering a range of indices when assessing overall fit. These indices included the χ^2^ goodness-of-fit index, comparative fit index (CFI), Tucker-Lewis index (TLI), and root mean square error of approximation (RMSEA). We considered values of >0.95 for CFI and TLI, and <0.05 for RMSEA to be indicative of a well-fitting model.

## Results

Skewness and kurtosis analyses conducted at the item- (i.e., indicator) level identified no problematic distributional properties across all latent variables. In our initial model, we specified two separate four-item latent social anxiety variables; one reflecting students’ concerns regarding their teacher and the other reflecting concerns regarding their classmates. However, fit indices and other relevant output indicated that a revised modeling approach may be necessary. In particular, the model did not appear to be well-fitting, χ^2^(1155) = 2136.39, *p* < 0.001, CFI = 0.92, TLI = 0.91, and RMSEA = 0.050 (90% confidence interval 0.047–0.053). In addition, the standardized residual covariance between students’ peer- and teacher-focused social anxiety perceptions (when treated as separate variables) was 0.87 (*p* < 0.001, 95% CI 0.79, 0.95), indicating a degree of redundancy between these constructs, and highlighting that (at least in an empirical sense) the specification of separate social anxiety variables was not justified.

Accordingly, we created a re-specified model (consistent with **Figure 1**) in which we made a number of modifications based on our initial analyses. First, we collapsed students’ social anxiety perceptions into a single (eight-indicator) latent variable. Second, on the basis of modification indices regarding the measurement portion of our initial model, we attempted to optimize model fit by relaxing error covariances where appropriate. We adopted this approach in line with Meehl’s (1990) assertion that, at some level, all variables are related to all others, and this process is also consistent with the theorized relations that exist between the variables included in this model. We incorporated 19 feasible modifications to the measurement portion of the model, by specifying error covariances between selected indicators within some latent variables (e.g., one covariance pathway was estimated among peer relatedness support indicators, four were estimated among peer RISE indicators). Following these modifications, we observed an improvement in fit indices, and with the exception of the significant chi-square value, fit indices collectively indicated a relatively well-fitting model, χ^2^(1146) = 1694.73, *p* < 0.001, CFI = 0.96, TLI = 0.95, and RMSEA = 0.038 (90% confidence interval 0.034–0.041). An overview of the various direct and indirect structural pathways within this model is provided in the following sections, and composite-level descriptive statistics and zero-order correlations between all variables are presented in **Table 1**.

**Table 1:** Aggregate-level descriptive data and correlations for all variables.

Variable	*M* (SD)	2	3	4	5	6	7	8	9	10	11	12
(1) Baseline LTPA	53.21 (37.56)	-0.19	-0.04	0.10	0.17	0.13	0.09	0.15	0.16	-0.06	-0.17	0.03
(2) Grade level	–	–	-0.02	0.03	-0.12	-0.13	-0.12	-0.02	-0.29	0.22	0.04	-0.15
(3) Teacher R-S	5.77 (1.24)		–	0.44	0.54	0.47	0.76	0.45	0.42	-0.21	-0.19	0.15
(4) Peer R-S	5.37 (1.27)			–	0.42	0.50	0.45	0.72	0.33	-0.16	-0.32	0.17
(5) Teacher RISE	4.07 (0.62)				–	0.73	0.58	0.47	0.66	-0.31	-0.33	0.28
(6) Peer RISE	3.98 (0.68)					–	0.53	0.53	0.57	-0.23	-0.30	0.24
(7) Teacher RNS	5.83 (1.19)						–	0.62	0.56	-0.25	-0.27	0.21
(8) Peer RNS	5.47 (1.24)							–	0.50	-0.22	-0.36	0.23
(9) Autonomous motivation	16.69 (3.67)								–	-0.34	-0.29	0.32
(10) Controlled motivation	9.62 (4.21)									–	0.44	-0.17
(11) Social anxiety	2.29 (1.00)										–	-0.14
(12) Engagement	5.63 (1.22)											–

Descriptives and correlations calculated using aggregate-level data within SPSS version 21. R-S, relatedness support; RISE, relation-inferred self-efficacy; RNS, relatedness need satisfaction. R-S measured 1–7, RISE 1–5, and RNS 1–7, where higher scores denote more positive perceptions. Higher scores for autonomous and controlled motivation denote stronger endorsement of that motivational orientation (possible range 3–21); Social anxiety, anxiety related to interactions with teacher and peers in PE (measured 1–5, where higher scores denote greater concern); Baseline LTPA, leisure-time physical activity (higher scores denote greater activity levels). *r* ≥ | 0.11| = *p* < 0.05; *r* ≥ | 0.14| = *p* < 0.01; *r* ≥ | 0.19| = *p* < 0.001.

### Direct Effects

Significant positive pathways indicated that students reported stronger relatedness need satisfaction regarding their teacher when they felt that their teacher (a) engaged in relatedness-supportive (i.e., interpersonally involving) behaviors, and (b) believed strongly in their (i.e., the student’s) ability (see **Table 2**). In addition, students reported more positive assessments of their teacher’s confidence in their ability when they felt that their teacher displayed relatedness-supportive instructional practices. We observed the same pattern of significant positive effects with regard to peer-related perceptions. In particular, students reported feeling more connected to their classmates when they felt that their classmates displayed relatedness-supportive behaviors, and when their classmates, as a whole, believed strongly in their (i.e., the student’s) ability. In addition, students tended to report that their classmates believed strongly in their ability when they felt that their classmates displayed relatedness-supportive behaviors.

**Table 2:** Standardized output for all structural pathways (unstandardized estimate in parentheses).

Pathway	Estimate	SE	*p*	95% CI
**Directional pathways**
Teacher R-S → Teacher RISE	0.42 (0.18)	0.066	<0.001	0.29, 0.55
Peer R-S → Peer RISE	0.42 (0.20)	0.061	<0.001	0.30, 0.54
Teacher R-S → Teacher RNS	0.63 (0.58)	0.082	<0.001	0.47, 0.80
Peer R-S → Peer RNS	0.63 (0.58)	0.053	<0.001	0.52, 0.73
Teacher RISE → Teacher RNS	0.24 (0.52)	0.076	0.001	0.10, 0.39
Peer RISE → Peer RNS	0.20 (0.40)	0.046	<0.001	0.11, 0.30
Teacher RISE → Autonomous motivation	0.50 (3.44)	0.093	<0.001	0.32, 0.68
Peer RISE → Autonomous motivation	-0.01 (-0.08)	0.063	0.830	-0.14, 0.11
Teacher RNS → Autonomous motivation	0.14 (0.47)	0.062	0.022	0.02, 0.26
Peer RNS → Autonomous motivation	0.19 (0.60)	0.051	<0.001	0.09, 0.29
Teacher RISE → Controlled motivation	-0.26 (-2.15)	0.092	0.004	-0.44, -0.08
Peer RISE → Controlled motivation	0.04 (0.32)	0.074	0.556	-0.10, 0.19
Teacher RNS → Controlled motivation	-0.01 (-0.01)	0.091	0.972	-.18, 0.18
Peer RNS → Controlled motivation	-0.12 (-0.44)	0.083	0.163	-0.28, -0.05
Teacher RNS → Social anxiety	-0.03 (-0.02)	0.078	0.742	-0.18, 0.13
Peer RNS → Social anxiety	-0.17 (-0.13)	0.081	0.040	-0.33, -0.01
Teacher RISE → Social anxiety	-0.01 (-0.01)	0.071	0.993	-0.14, 0.14
Peer RISE → Social anxiety	-0.09 (-0.13)	0.060	0.153	-0.20, 0.03
Autonomous motivation → Social anxiety	-0.02 (-0.01)	0.080	0.824	-0.17, 0.14
Controlled motivation → Social anxiety	0.37 (0.08)	0.053	<0.001	0.27, 0.47
Teacher RISE → Engagement	0.10 (0.24)	0.111	0.375	-0.12, 0.32
Peer RISE → Engagement	0.05 (0.11)	0.083	0.541	-0.11, 0.21
Autonomous motivation → Engagement	0.21 (0.08)	0.079	0.007	0.06, 0.37
Controlled motivation → Engagement	-0.02 (-0.01)	0.063	0.690	-0.15, 0.10
**Covariances/residual covariances**
Teacher R-S ↔ Peer R-S	0.50 (0.69)	0.070	<0.001	0.37, 0.64
Teacher RISE ↔ Peer RISE	0.67 (0.15)	0.060	<0.001	0.55, 0.79
Teacher RNS ↔ Peer RNS	0.46 (0.22)	0.087	<0.001	0.29, 0.63

R-S, relatedness support; RISE, relation-inferred self-efficacy; RNS, relatedness need satisfaction; RAI, relative autonomy index (higher scores denote greater autonomous relative to controlled motivation); Social anxiety, anxiety related to interactions with teacher/peers in PE. Standardized estimates interpreted in line with Cohen’s (1992) recommended effect size criteria (i.e., 0.10 = small, 0.30 = moderate, 0.50 = large). With the exception of anxiety, higher scores denote more positive perceptions. Variance explained: teacher RISE = 22%; Peer RISE = 20%; Teacher RNS = 62%; Peer RNS = 56%; Autonomous motivation = 53%; Controlled motivation = 14%; Social anxiety = 25% (all *p* < 0.001); Engagement = 13% (*p* = 0.015).

Significant pathways emerged for three of the four interpersonal perception variables that were hypothesized to predict students’ autonomous motivation for PE. No significant effect was apparent for peer-focused RISE; however, students did report greater autonomous motivation for PE when they believed that their PE teacher was highly confident in their ability (i.e., teacher-focused RISE). Analyses also revealed that students reported greater autonomous motivation for their participation in PE when they felt valued, understood by, and close to, their classmates (i.e., peer-focused relatedness need satisfaction) and their teacher (i.e., teacher-focused relatedness need satisfaction). It is worth noting that these effects upon autonomous motivation occurred over and above the effects of students’ grade level and baseline LTPA (see **Table 3** for covariate pathways). Only one significant pathway emerged for the variables specified as potential predictors of controlled motivation; that is, students reported greater controlled motivation when they believed that their teacher was not highly confident in their ability in PE.

**Table 3:** Standardized output for all covariate pathways specified within the model (unstandardized estimate in parentheses).

Effect	Estimate	SE	*p*	95% CI
LTPA → Teacher RISE	0.19 (0.01)	0.076	0.013	0.04, 0.34
LTPA → Peer RISE	0.10 (0.01)	0.062	0.095	-0.02, 0.22
LTPA → Teacher RNS	0.07 (0.01)	0.034	0.036	0.01, 0.14
LTPA → Peer RNS	0.08 (0.01)	0.042	0.051	0.00, 0.17
LTPA → Autonomous motivation	-0.02 (-0.01)	0.032	0.546	-0.08, 0.04
LTPA → Controlled motivation	0.04 (0.01)	0.039	0.275	-0.03, 0.12
LTPA → Social anxiety	-0.12 (-0.01)	0.060	0.046	-0.24, -0.01
LTPA → Engagement	-0.05 (-0.01)	0.056	0.370	-0.16, 0.06
Grade → Teacher RISE	-0.07 (-0.03)	0.045	0.101	-0.16, 0.01
Grade → Peer RISE	-0.10 (-0.05)	0.041	0.014	-0.18, -0.02
Grade → Teacher RNS	-0.09 (-0.08)	0.058	0.124	-0.20, 0.02
Grade → Peer RNS	0.01 (0.01)	0.059	0.964	-0.11, 0.12
Grade → Autonomous motivation	-0.23 (-0.70)	0.044	<0.001	-0.32, -0.15
Grade → Controlled motivation	0.22 (0.79)	0.055	<0.001	0.11, 0.33
Grade → Social anxiety	-0.07 (-0.05)	0.043	0.114	-0.15, 0.02
Grade → Engagement	-0.09 (-0.10)	0.147	0.526	-0.38, 0.20

LTPA, leisure-time physical activity; Social anxiety, anxiety related to interactions with teacher and peers in PE. ‘Grade’ denotes the academic grade/year of the student (i.e., grade/years 7–10). For example, analyses indicated that as grade level increased (i.e., for older students), autonomous motivation decreased.

Alongside the abovementioned covariates, we specified six potential predictors of students’ social anxiety in PE (i.e., both forms of motivation, relatedness need satisfaction, and RISE), and observed significant effects for two of these variables. First, when students reported favorable perceptions of peer-focused relatedness need satisfaction (i.e., when they felt a close connection to their PE classmates), this aligned with lower levels of anxiety regarding the way in which their teacher/peers evaluated them in PE. Second, students reported greater social anxiety when they scored highly on controlled motivation for PE. As shown in **Table 2**, significant pathways did not emerge in relation to social anxiety for teacher-based relatedness need satisfaction, autonomous motivation, or students’ RISE inferences. In terms of the remaining in-class outcome within our model, aside from the covariate pathways we modeled for grade level and baseline LTPA, we specified four potential predictors of students’ behavioral engagement in PE. Although significant pathways were not observed for controlled motivation or either RISE variable, students who reported strong autonomous motivation for PE were rated by their teacher as displaying greater in-class engagement.

### Indirect Pathways

We requested estimates of all possible specific indirect effects between students’ interpersonal perceptions and the variables that we specified as the most distal in-class outcomes (i.e., engagement, social anxiety). In terms of students’ behavioral engagement, we observed a number of significant indirect pathways associated with both teacher- and peer-focused appraisals. With respect to teacher-focused effects, the most detailed pathway revealed a positive indirect relationship linking students’ perceptions of their teacher’s relatedness-support with their engagement, via favorable teacher-focused RISE, relatedness need satisfaction, and autonomous motivation (i.e., teacher-derived relatedness support → teacher-focused RISE → teacher-based relatedness need satisfaction → autonomous motivation → engagement; standardized estimate = 0.003, SE = 0.002, *p* = 0.044, 95% CI 0.001, 0.006). Three smaller indirect chains within this pathway were also significant; one that originated with students’ perceptions of relatedness-support from their teacher, and that was identical aside from the exclusion of relatedness need satisfaction (i.e., teacher-derived relatedness support → teacher-focused RISE → autonomous motivation → engagement; standardized estimate = 0.045, SE = 0.022, *p* = 0.045, 95% CI 0.001, 0.09), and another that originated with students’ RISE appraisals regarding their teacher (i.e., teacher-focused RISE → autonomous motivation → engagement; standardized estimate = 0.106, SE = 0.048, *p* = 0.028, 95% CI 0.012, 0.201).

We observed a similar pattern with respect to peer-focused appraisals in relation to engagement, whereby peer-focused appraisals displayed positive indirect effects through favorable autonomous motivation scores. The first pathway originated with students’ peer-focused RISE appraisals, and aligned positively with engagement through relatedness need satisfaction and autonomous motivation (i.e., peer-focused RISE → peer-focused relatedness need satisfaction → autonomous motivation → engagement; standardized estimate = 0.008, SE = 0.004, *p* = 0.041, 95% CI 0.001, 0.016). A second pathway was similar in nature, but originated with students’ perceptions of peer-derived relatedness support (i.e., peer-derived relatedness support → peer-focused relatedness need satisfaction → autonomous motivation → engagement; standardized estimate = 0.025, SE = 0.011, *p* = 0.020, 95% CI 0.004, 0.047), and a final, shorter pathway was also apparent that linked peer-focused relatedness need satisfaction with engagement, via autonomous motivation (i.e., peer-focused relatedness need satisfaction → autonomous motivation → engagement; standardized estimate = 0.04, SE = 0.018, *p* = 0.021, 95% CI 0.006, 0.075).

Three indirect pathways linked students’ interpersonal appraisals with their social anxiety, again originating from teacher- as well as peer-focused appraisals. In terms of students’ perceptions regarding their teacher, the most intricate pathway linked favorable perceptions of teacher-derived relatedness support with lower social anxiety, via teacher-focused RISE and controlled motivation (i.e., teacher-derived relatedness support → teacher-focused RISE → controlled motivation → social anxiety; standardized estimate = -0.041, SE = 0.015, *p* = 0.005, 95% CI -0.070, -0.012). A shorter pathway within this chain, originating at teacher-focused RISE, was also significant (i.e., teacher-focused RISE → controlled motivation → social anxiety; standardized estimate = -0.097, SE = 0.038, *p* = 0.011, 95% CI -0.172, -0.022). A single indirect pathway emerged for peer-focused perceptions, and demonstrated a negative link between peer-derived relatedness support and social anxiety, via peer-focused relatedness need satisfaction (i.e., peer-derived relatedness support → peer-focused relatedness need satisfaction → social anxiety; standardized estimate = -0.105, SE = 0.050, *p* = 0.034, 95% CI -0.202, -0.008).

## Discussion

Researchers have demonstrated that students’ interpersonal interactions and perceptions are important in shaping their experiences in PE (e.g., Cox et al., 2009). Although much of this work has been couched within SDT (see Ntoumanis, 2012), a growing body of evidence also supports the utility of the tripartite efficacy framework for studying relational processes in this setting (e.g., Bourne et al., 2015). To date though, studies using these models have focused primarily on students’ perceptions about their teachers, and as a result, our understanding is somewhat limited regarding the interplay between (and independent implications of) students’ peer- and teacher-related appraisals (cf. Standage and Emm, 2014). By integrating concepts rooted in SDT and the tripartite efficacy model, we explored the relations between a range of teacher- and peer-focused interpersonal variables, as well as their predictive effects with respect to important in-class outcomes. Analyses demonstrated complementary relations between distinct relational perceptions, and revealed that a number of these variables aligned (directly and/or indirectly) with downstream outcomes.

Focusing first on the interpersonal predictors within our model, we observed support for the distinguishability of teacher- and peer-related perceptions. Although it is well-established that the perception of need-supportive behaviors acts as a precursor to need satisfaction (e.g., Standage et al., 2005), this study demonstrated that these effects are apparent when modeled separately for distinct social ‘agents’ (i.e., teachers *and* peers) within this context. For example, students reported greater relatedness need satisfaction from their peers and from their teacher when these agents were independently deemed to engage in highly relatedness-supportive behaviors. The emergence of these direct effects was consistent with tenets of SDT; however, we also observed *indirect* relations between need support and need satisfaction (for both teacher- and peer-related perceptions) that held relevance for the integration of SDT and relational efficacy concepts. In particular, students reported more favorable RISE appraisals when they believed that the focal agent (i.e., teacher or peers) engaged in relatedness-supportive behaviors, which in turn predicted enhanced relatedness need satisfaction regarding that agent. These findings highlight that RISE beliefs may act as a perceptual mechanism that (in part) supports the link between relatedness support and relatedness need satisfaction. Indeed, it seems plausible that the care and individualized attention that characterize relatedness-supportive interactions may encourage students to believe that the providers of such behaviors believe strongly in their ability, which subsequently fosters perceptions of closeness and support with respect to the provider (cf. Lent and Lopez, 2002).

The predictive effects that we observed in relation to students’ autonomous motivation underscored the importance of incorporating distinct teacher- and peer-focused assessments. Specifically, students endorsed stronger autonomous (i.e., enjoyment, interest, value) motives for PE when they felt close to, and supported by, their teacher *and* their classmates. The role of relatedness need satisfaction in relation to autonomous motivation is well-established within SDT (see Ryan and Deci, 2008), and has been demonstrated previously in this context (e.g., Standage et al., 2006; Cox et al., 2009). To our knowledge, though, these findings are novel inasmuch as teacher- and peer-derived relatedness perceptions each uniquely contributed to adaptive motivational processes. It was also noteworthy that even when controlling for the predictive effects associated with relatedness need satisfaction, students’ teacher-focused RISE estimations also emerged as a significant (positive) predictor of autonomous motivation and (negative predictor of) controlled motivation. The relationship with autonomous motivation is consistent with theorizing by Lent and Lopez (2002), who contended that favorable RISE inferences may promote responses that are either directly associated with (e.g., enjoyment), or implicated in the promotion of (e.g., elevated perceptions of one’s own competence), adaptive motivational processes. There is no prior empirical evidence to substantiate the relationship that was observed between teacher-focused RISE and controlled motivation; nonetheless, it is interesting that believing that one’s teacher is confident in one’s ability may not only promote greater autonomous motives, but may also assist in reducing feelings of pressure and obligation associated with participation in PE.

In future, it would be worthwhile to examine whether the motivational effects we observed for RISE remain when controlling for other SDT-based (e.g., autonomy need satisfaction) and tripartite efficacy (e.g., other-efficacy) predictors. Indeed, existing tripartite efficacy work in PE has demonstrated non-significant direct pathways between RISE and autonomous motivation (Jackson et al., 2013) when controlling for other relevant efficacy perceptions, and we did not observe a significant pathway between students’ peer-focused RISE and either motivational index. That being the case, future research that examines motivation for different focal activities or sub-domains (within a given context) may provide some insight into the differing nature of the RISE effects that we observed in this investigation. For example, it may emerge that favorable RISE appraisals regarding one’s classmates assist specifically in promoting motivation regarding one’s participation in group-based activities performed with one’s classmates in PE (rather than with respect to one’s participation in PE in general). There may also be additional explanatory mechanisms that were unmeasured in this investigation, and that may moderate the effects of RISE (and/or differentially moderate the effects of distinct variants of RISE) appraisals upon motivation and other outcomes (cf. Lent and Lopez, 2002). For instance, one’s RISE inferences may be particularly salient when (a) the target of the inference holds a position of authority (e.g., a teacher in comparison to one’s peers), (b) the perceiver strongly identifies with the target individual/group, and/or (c) the perceiver has limited experience, lacks resilience, or has a limited capacity to accurately appraise his/her own ability.

Despite observing support for three out of the four variables that we specified as predictors of autonomous motivation, it is worth noting that neither teacher- nor peer-focused relatedness need satisfaction emerged as a negative predictor of controlled motivation, as was originally hypothesized. We anticipated that satisfaction of one’s need for relatedness would be responsible for a process of internalization, whereby individuals reported lower controlled motivation at the same time as heightened autonomous motivation. It is possible that by separating our assessment of relatedness need satisfaction (rather than using a global index), though, we may have diluted the relative effect of these predictors in relation to controlled motivation. Alternatively, it is also possible that while relatedness need satisfaction was strongly related to autonomous motivation, its effect on controlled motivation may be influenced by one or more unmeasured moderator variables. For example, for some individuals (e.g., those who hold a stable and positive self-image), feeling close to one’s teacher and classmates may alleviate feelings of coercion and pressure, but for others (e.g., those who tend to be more concerned with impression management), such appraisals may engender the feeling that one has to ‘live up to’ the standards of others, and must not let those individuals down. In future, by continuing to model autonomous and controlled motivation separately, it would be intriguing to examine the factors that may shape the direction and magnitude of the effects we explored.

Alongside our focus on motivational processes, we also gained insight into students’ engagement and social anxiety in PE. Perhaps the most noteworthy direct predictive effects that emerged for both of these outcomes stemmed from students’ motivation. In particular, students who strongly endorsed controlled motives for their participation in PE reported higher levels of concern regarding the way in which they were evaluated by their teacher and peers. Those who endorse controlled motives are concerned with others’ impressions, adhering to rules, and avoiding sanction, and so it would be expected that those individuals may also be disposed to greater concern regarding their interactions within PE. In terms of direct effects, students also reported lower social anxiety when they felt supported by, and connected to, their classmates (cf. Cox et al., 2009). More robust (e.g., experimental) insight is necessary to draw causal conclusions regarding this pathway; nonetheless, this effect provides some support for the potential anxiolytic effects that may be derived by implementing strategies that promote inclusivity among classmates in PE. With respect to engagement, we observed that students were rated more positively by their teacher when they reported strong autonomous motivation toward PE. Although support for similar engagement-related findings has been provided previously (e.g., Ntoumanis, 2005), the role of autonomous motivation in supporting relationships between interpersonal appraisals and engagement was a novel contribution of this study. Indeed, aside from direct effects, our analyses demonstrated that students’ interpersonal perceptions were linked with in-class outcomes (i.e., engagement and social anxiety) via indirect pathways that operated primarily through their motivation. For example, favorable relatedness support and RISE perceptions relating to one’s teacher and peers were indirectly associated with greater engagement and lower social anxiety ratings, through a series of pathways that incorporated autonomous or controlled motivation. Taken together, these indirect effects offer some (albeit observational) insight into the mechanisms through which relational perceptions might support students’ PE experiences.

When reflecting on the contribution of the study, it is necessary to consider important design limitations and accompanying future research directions. First, we examined a restricted group of the predictors outlined in SDT and the tripartite efficacy framework (i.e., relatedness-based perceptions and RISE), and future work is encouraged that addresses this issue by accounting for additional social processes (e.g., autonomy-support, structure, other-efficacy). Second, we considered only the behavioral aspect of student engagement, and did not account for important emotional and cognitive dimensions. In future, in seeking to more fully understand student behavior within PE, it would be worthwhile to examine interpersonal appraisals alongside a more comprehensive assessment of engagement processes. Third, it is also important to acknowledge that our data were obtained from females-only and from only one school, and so we urge caution when gaging the generalizability of our findings. Further work that tests these pathways with a more diverse sample (in both a geographic and socioeconomic sense), and that examines the extent to which these relationships hold across gender, would be advised.

Although we developed a structural model on the basis of theory and research, we are also unable to derive insight into causal processes with this design. Accordingly, investigators are encouraged to draw from existing methods for manipulating these relational ‘predictors’ (cf. Tessier et al., 2010) to examine the utility of teacher- and/or peer-mediated interventions for bolstering in-class outcomes. Similarly, designs that utilize repeated assessments (e.g., cross-lagged, longitudinal) would allow for insight into the potential bi-directional nature of the relations that we specified in our investigation. Finally, our primary variables were assessed at the student level, and future work would be worthwhile that adopts a multilevel perspective to the study of interpersonal relations within PE (cf. Taylor and Ntoumanis, 2007). For example, it would be enlightening to obtain assessments of teacher instructional behavior (e.g., through recordings and expert coding; Haerens et al., 2013) and peer-derived motivational climate, with the goal of understanding the class-level conditions under which favorable relational perceptions develop among students.

In summary, this investigation offered insight into a novel network of teacher- and peer-focused perceptions within PE. With respect to conceptual innovation, this study demonstrated support for the direct and indirect relations between SDT-based concepts, relational efficacy perceptions, and salient in-class processes. Meanwhile, from a practical perspective, the indirect pathways that emerged in our investigation provide insight into the potential ways through which supportive social interactions may support adaptive in-class outcomes. Accordingly, practitioners and teachers may focus their attention on fostering positive PE experiences through providing support for the development of favorable interpersonal perceptions. In doing so, strategies that encourage teachers and students to display interest, warmth, and supportive (i.e., RISE-enhancing; see, for example, Saville et al., 2014) feedback may be valuable in promoting desired behavioral and affective states within PE. In closing, the challenges associated with creating high-quality PE environments among this population group are well documented (e.g., Dwyer et al., 2006), and these findings demonstrate that one avenue for stimulating positive experiences may be through optimizing students’ interpersonal interactions with teachers and peers.

## Conflict of Interest Statement

The authors declare that the research was conducted in the absence of any commercial or financial relationships that could be construed as a potential conflict of interest.

## Footnotes

1 The ‘combined’ internal consistency is reported here in light of the modeling strategy that was adopted in our final analyses (i.e., combining teacher and peer items within a single latent variable). The rationale for combining teacher and peer perceptions is outlined in the results section.
